# Ge quantum dot arrays grown by ultrahigh vacuum molecular-beam epitaxy on the Si(001) surface: nucleation, morphology, and CMOS compatibility

**DOI:** 10.1186/1556-276X-6-522

**Published:** 2011-09-05

**Authors:** Vladimir A Yuryev, Larisa V Arapkina

**Affiliations:** 1 A. M. Prokhorov General Physics Institute of RAS, 38 Vavilov Street, 119991 Moscow, Russia

## Abstract

Issues of morphology, nucleation, and growth of Ge cluster arrays deposited by ultrahigh vacuum molecular beam epitaxy on the Si(001) surface are considered. Difference in nucleation of quantum dots during Ge deposition at low (≲600*°*C) and high (≳600*°*C) temperatures is studied by high resolution scanning tunneling microscopy. The atomic models of growth of both species of Ge huts--pyramids and wedges-- are proposed. The growth cycle of Ge QD arrays at low temperatures is explored. A problem of lowering of the array formation temperature is discussed with the focus on CMOS compatibility of the entire process; a special attention is paid upon approaches to reduction of treatment temperature during the Si(001) surface pre-growth cleaning, which is at once a key and the highest-temperature phase of the Ge/Si(001) quantum dot dense array formation process. The temperature of the Si clean surface preparation, the final high-temperature step of which is, as a rule, carried out directly in the MBE chamber just before the structure deposition, determines the compatibility of formation process of Ge-QD-array based devices with the CMOS manufacturing cycle. Silicon surface hydrogenation at the final stage of its wet chemical etching during the preliminary cleaning is proposed as a possible way of efficient reduction of the Si wafer pre-growth annealing temperature.

## Introduction: Background and problem statement

Heteroepitaxial Ge/Si and SiGe/Si structures are among the most promising materials of modern nanoelectronics and nanophotonics [1-12]. Lately industry has developed numerous radiofrequency devices on the basis of SiGe/Si structures with bands wider than 100 GHz, which already compete with GaAs-based components. Except that, SiGe-based technology has allowed one to approach to the development of the most important elements of single-crystalline integrated microphotonics-- laser diodes and detectors for fiberoptic communications; SiGe-based waveguides are already available. So, the forecasted forthcoming breakthrough would enable the solution of two important problems--(i) development of monolithic VLSI circuits for fiberoptic telecommunications and (ii) replacement of electronic data buses by optical ones. Additionally, encouraging results have been recently obtained in development of emitting THz and mid-infrared devices based on SiGe/Si heterostructures. And finally, application of Ge/Si and SiGe/Si hetersructures might enable a breaking progress in IR imaging technology opening a way to creation of multispectral photodetector arrays integrated with readout circuitry on a single-crystalline chip.

Dense arrays of Ge quantum dots (QD) are of importance to all practically significant applications in optoelectronics and microelectronics. QD array is usually referred to as dense array if interactions among adjacent clusters play an important role [13], i.e., a tunnel coupling between Ge clusters arise [14]; such arrays should be considered as a whole in terms of behavior of current carrier and transport properties [10]. Ge/Si hetrostructure can include both isolated QD arrays, i.e., arrays which do not mutually interact and separated by a thick enough (*>*30 nm) Si layer, and superlattices of QD arrays, in which arrays are separated by thin Si layers and which, like SiGe/Si superlattices with quantum wells, represent a single coupled system. In addition, it is known that, if distances between layers of Ge clusters are as small as a few nanometers in the direction of the structure growth, ordering of Ge clusters is observed in this direction [15]; they form chains which can be composed by tens of clusters if a number of layers is large [16]. Like atoms, Ge clusters form a sort of molecules in which electron density redistributes among clusters depending on distances between them (thicknesses of Si barrier layers), as if changing a type of chemical bond from covalent to ionic. This phenomenon opens a wide perspective to designing heterostructures with various optical and electrical properties.

Recently, an interest of researchers has been attracted by heterostructures with ordered arrays of quantum dots, in particular, by ordered arrays of Ge nanoclusters in the Si matrix [17]. An idea of formation of a QD array, which would combine advantages of a single QD with benefits of a dense array, seems to be the most promising.^a ^Controllable ordering of clusters in all three directions would enable creation of a volumetric crystal in which QDs play a role of atoms. In such artificial crystal, unique opportunities appear to designing wave functions of carriers by filling corresponding quantum states of QDs by electrons or holes (like it is the case for *s*, *p*, and *d *atomic configurations). As opposed to impurity states in semiconductors, dense 3D array of QDs would be an ensemble of multicharged centers in which an essential role would be played by the Coulomb potential. A concept of the QD crystal, which is considered as a 3D lattice of artificial atoms, implies a new material with spatial ordering on the scale comparable with the de Broglie wave-length for electrons. Non-locality of the quantum-mechanical bonding together with the Coulomb interaction of carriers localized in close QDs may result in new optical and electronic properties arising from the collective nature of electronic states. In contrast to stochastically located QDs, in this case, these properties would not be averaged over components of a crystal. Main properties of such ensemble would reproduce peculiarities inherent to ordinary solids, such as appearance of two-dimentional or three-dimentional minibands, separated by minigaps, in lieu of localized quantum states intrinsic to separate QDs. It is necessary for QD crystal that QDs would be ordered to precise periodicity, the sizes of QDs would be equal, and distances between QDs would be small enough for wave functions to overlap.

Such QD crystals would be very prospective for application in nanoelectronics, spintronics and, likely, in quantum computing, as well as in devices of silicon optoelectronics such as highly efficient sources and detectors of infrared and terahertz emission enabling integration to silicon VLSI circuitry.

Main restriction for use of such Ge/Si heterostructures with dence arrays of self arranged Ge QDs is associated with the spread sizes of Ge clusters and their tendency to disordering on the surface. Both these factors cause tailing of a discrete spectrum. Additional difficulty is the necessity for all technological steps to be embedded into VLSI manufacturing process or, in other words, meet requirements of CMOS compatibility.

To be able to accomplish the above ambitious task, a deep knowledge of physical processes on silicon surface during its preparation and in germanium and silicon films during the heterostructure formation is strongly required. This article represents some results of our recent investigations in this direction.

## Methods

### Equipment

The experiments were carried out using an integrated ultrahigh vacuum instrument [18-20] built on the basis of the Riber SSC 2 surface science center with the EVA 32 molecular-beam epitaxy (MBE) chamber equipped with the RH20 reflection high-energy electron diffraction (RHEED) tool (Staib Instruments) and connected through a transfer line to the STM GPI-300 ultrahigh vacuum scanning tunneling microscope [21-23].^b ^A preliminary annealing and outgassing chamber is also available in the instrument.

The pressure of about 5 × 10^-9 ^Torr was kept in the preliminary annealing chamber. The MBE chamber was evacuated down to about 10^-11 ^Torr before processes; the pressure increased to nearly 2 × 10^-9 ^Torr at most during the sample surface deoxidization process and 10^-9 ^Torr during Ge or Si deposition. The residual gas pressure did not exceed 10^-10 ^Torr in the STM chamber.

The instrument enables the STM study of samples at any stage of Si surface preparation and MBE growth. The samples can be consecutively moved into the STM chamber for the analysis and back into the MBE vessel for further treatments or Ge, Si, or SiGe deposition as many times as required never leaving the UHV ambient and preserving the required cleanness for MBE growth and STM investigations with atomic resolution. RHEED experiments can be carried out in situ, i.e., directly in the MBE chamber during a process [19].

Sources with the electron beam evaporation were used for Ge or Si deposition. The deposition rate and coverage were measured using the Inficon Leybold-Heraeus XTC 751-001-G1 film thickness monitor equipped with the graduated in-advance quartz sensors installed in the MBE chamber. Tantalum radiators were used for sample heating from the rear side in both preliminary annealing and MBE chambers. The temperature was monitored with chromel-alumel and tungsten-rhenium thermocouples of the heaters in the preliminary annealing and MBE chambers, respectively. The thermocouples were mounted in vacuum near the rear side of the samples and in situ graduated beforehand with respect to the IMPAC IS 12-Si pyrometer that measures the sample temperature through chamber windows. The temperature distribution uniformity over a surface was also investigated in advance; the deviations from mean values were found to be within ±3°C for the half-radius areas around the centers of 2" wafers over the whole temperature interval applied in this study.

The composition of residual atmosphere in the MBE camber was monitored using the SRS RGA-200 residual gas analyzer before and during the process.

The STM tip was *ex situ *made of the tungsten wire and cleaned by ion bombardment [24] in a special UHV chamber connected to the STM one.

In this work, the images were obtained in the constant tunneling current (*I_t_*) mode at the room temperature. The STM tip was zero-biased while the sample was positively or negatively biased (*U*_s_) when scanned in empty- or filled-states imaging mode.

Original firmware [21-23] was used for data acquisition; the STM images were processed afterward using the WSxM software [25].

### Sample preparation procedures

#### Preparation of samples with deposited Ge layers

Initial samples for STM were 8 × 8 mm^2 ^squares cut from the specially treated commercial boron-doped Czochralski-grown (CZ) Si(100) wafers (*p*-type, *ρ *= 12 Ωcm). After washing and chemical treatment following the standard procedure described elsewhere [26] (which included washing in ethanol, etching in the mixture of HNO_3 _and HF, and rinsing in the deionized water [27]), the silicon substrates were mounted on the molybdenum STM holders and inflexibly clamped with the tantalum fasteners. The STM holders were placed in the holders for MBE made of molybdenum with tantalum inserts. Then, the substrates were loaded into the airlock and transferred into the preliminary annealing chamber where they were outgassed at the temperature of around 565°C for more than 6 h. After that, the substrates were moved for final treatment and Ge deposition into the MBE chamber where they were subjected to two-stages annealing during heating with stoppages at 600°C for 5 min and at 800°C for 3 min [18]. The final annealing at the temperature greater than 900°C was carried out for nearly 2.5 min with the maximum temperature of about 925°C (1.5 min). Then, the temperature was rapidly lowered to about 750°C. The rate of the further cooling was around 0.4°C/s that corresponded to the 'quenching' mode applied in [19]. The surfaces of the silicon substrates were completely purified of the oxide film as a result of this treatment [19,28,29].

Ge was deposited directly on the deoxidized Si(001) surface. The deposition rate was varied from about 0.1 to 0.15 Å/s; the effective Ge film thickness (*h*_Ge_) was varied from 3 to 18 Å for different samples. The substrate temperature during Ge deposition (*T*_gr_) was 360 or 530°C for the low-temperature mode and 600 or 650°C for the high-temperature mode. The rate of the sample cooling down to the room temperature was approximately 0.4°C/s after the deposition.

After cooling, the prepared samples with Ge layers were moved for analysis into the STM chamber.

#### Preparation of samples for Si(001) surface analysis

Wafers for Si(001) surface analysis by STM and RHEED were the same as for Ge MBE. Initially, the specimens were chemically etched in the RCA etchant [30] and processed to form a surface terminated by hydrogen atoms (Si:H). The hydrogenated Si:H samples were prepared by etching in solutions containing HF at the final stage of the RCA process [31]. We used the following solutions with pH = 2, 4 or 7: a dilute HF solution (5 or 0.5%), buffered NH_4_F + HF or NH_4_F solutions. After that, the Si:H samples were pre-treated for 2 h at the temperature of ~300°C and the pressure of less than 5 × 10^-11 ^Torr in the MBE chamber. The second phase of the thermal treatment was conducted at the temperatures of 800, 650, 610, 570, 550, 530, or 470°C. Duration of this phase was chosen to a form of the RHEED pattern. The samples were quenched after heat treatments at the rate of ~0.4°C/s [19].

## Results and discussion

### Nucleation

#### Hut nucleation at low and high temperatures

**Figure 1:**
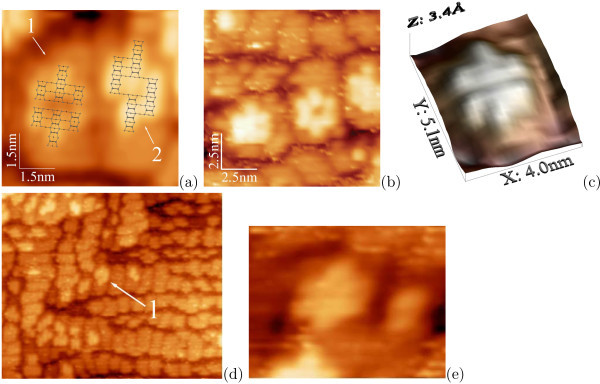
**STM empty-state images of hut nuclei on Ge WL formed at different temperatures**: **a **pyramid (1) and wedge (2) nuclei on the adjacent *M *× *N *patches of WL; *T*_gr _= 360°C, *h*_Ge _= 6 Å; the structural models [20,32] are superimposed on the corresponding images in **a; b, c **pyramid nuclei on WL formed at low temperature (*T*_gr _= 360°C): **b ***h*_Ge _= 5.4 Å; **c ***h*_Ge _= 6 Å; **d, e **a pyramid nucleus on WL formed at high temperature, *h*_Ge _= 5 Å: **d ***T*_gr _= 600°C, 43 × 37 nm; **e ***T*_gr _= 650°C, 7.8 × 6 nm.

*Nucleation of Ge clusters at low temperatures *has been a topic of numerous experimental and theoretical investigations for a number of years (see a brief review section in article [18]). Recently, we have described two characteristic formations composed by epitaxially oriented Ge dimer pairs and chains of four dimers on the wetting layer (WL) patches that were interpreted by us as two types of hut nuclei: an individual type for each species of huts--pyramids or wedges (Figure 1a-c) [18,20,32]. These nuclei are always observed to arise on sufficiently large WL patches: there must be enough room for a nucleus on a single patch; a nucleus cannot be housed on more than one patch [32].

Both types of the hut nuclei appeared to arise at the same WL *M *× *N *patch thickness [20,33], hence, at the same WL stress to relieve it. Therefore, they appear at the same strain energy (and with equal likelihoods, see Refs. [18,32]). This means that they are degenerate by the formation energy: if they had different formation energies they would appear at different WL thicknesses; the first of the types of huts, which nucleates on the surface, releases the stress; the second one never appears therefore. Hence, they can occur only simultaneously and their formation energies can be only equal.

Presently, we have no satisfactory explanation of this phenomenon and can only propose a very preliminary interpretation of the observed simultaneous appearance of the two kinds of nuclei on WL. The explanation is based on modeling of Ge cluster formation energy performed in Ref. [34]. Brehm et al. [34] have explored Ge island nucleation during MBE at much higher temperatures than those applied in this work; therefore, theoretical results of Ref. [34] describe the experimental data obtained for the case of the high-temperature growth mode, which differs considerably from the low-temperature one [18]. However, the modeling could also apply for the low-temperature growth. According to Ref. [34], flat Ge islands--in our case, nuclei and small huts--likely occur on WL because of an energy benefit which arises in exposing the compressed {105} facets, rather than in relaxing the volumetric elastic energy, as it takes place in the usual Stranski-Krastanov mechanism. At low temperatures, this effect may stabilize clusters, however, preventing their further ripening (this agrees with our observations presented recently in Ref. [18]). If this is the case, the actual volumetric and structural form of clusters likely does not impact very much in their formation energy.^c^

As distinct from the low-temperature mode, *Ge cluster nucleation at high temperatures *may go on in two ways. The first way is similar to the process of hut nucleation at low temperatures. Pyramids were observed to nucleate in such a way. Figure 1d, e illustrates this statement: the pyramid nuclei, absolutely the same as those observed in the samples grown at low temperature, are seen on the WL patches in the images of the samples obtained at *T*_gr _^= ^650°C. Their density was small, and they were mainly situated in the vicinity to large mature pyramids, which arise at early stages of Ge deposition and have much greater sizes than huts formed at low temperatures at the same values of *h*_Ge _[35]. The WL surface mainly consisted of monoatomic steps and narrow terraces in these areas (Figure 1d).

**Figure 2:**
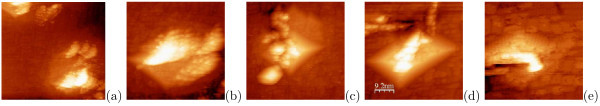
**Formation of {105} facets from shapeless areas with excess of Ge**: STM images of different phases of faceting, *T*_gr _= 650°C, *h*_Ge _= 5 Å; **a **a shapeless Ge 'heap' without faceting, 150 × 141 nm; **b **at the outset of faceting, 64 × 64 nm; **c**-**e **after growth stoppage and annealing at the growth temperature; **c **72 × 72 nm; **d **46 × 46 nm; **e **23 × 23 nm.

The second way, somewhat resembling the process described by Goldfarb et al. [36] for the case of the gas-source MBE (and thick hydrogenated WL), is illustrated by Figure 2. At small values of *h*_Ge_, regions containing excess of Ge atoms were observed on the surface. Usually, they were not resolved as structured formations and resembled shapeless heaps of Ge (Figure 2a). Pits usually accompanied them. Heap density was about 10^9 ^cm^-2^. Some of heaps had started to form the {105} facets during Ge deposition (Figure 2b).

Stoppage of Ge deposition and subsequent annealing at *T*_gr _resulted in formation of volumetric structures partially or even completely faceted by {105} planes, transforming 'heaps' to some similarities of huts (Figure 2c, d, e).

We have never observed such process at low temperatures of growth and suppose it to be inherent only to the high-temperature array formation mode.

#### Array nucleation and growth outset

Since the pioneering work by Mo et al. [37], it has been known that deposition of Ge on Si(001) beyond 3 ML (1 ML ≈ 1.4 Å) leads to formation of huts [37-39] on WL with high number density (≳10^10 ^cm^-2^, Refs. [18,20,40]). Some later the value of Ge coverage, at which 3D clusters emerged, was confirmed by Iwawaki et al. [41] who, in the course of a comprehensive STM study of the low-temperature epitaxial growth of Ge on Si(001) [41-44], directly observed appearance of minute (a few ML height) 3D Ge islands at 300°C on (*M *× *N*)-patched WL; deposition of 4 ML of Ge resulted in formation of a dense array of small huts. Various values of Ge coverage, at which the transition from 2D to 3D growth occurs, are presented in the literature. For example, an abrupt increase in hut density at the coverage of 3.16 ML was detected for Ge deposition at 300°C and 0.06 ML/min [40]. A detailed phase diagram of the Ge film on Si(001) derived from experiments carried out by recording RHEED gave the coverages corresponding to the "2D-to-hut" transition from ~2.5 to ~3 ML for the growth temperature interval from 300 to 400°C (and different values for different temperatures) [45]. Photoluminescence study of Ge huts deposited at the temperature of 360°C showed that evolution from "quantum-well-like" (attributed to WL) to "quantum-dot-like" (attributed to Ge huts) emission occurred at a coverage of ~4.7 ML in PL spectra obtained at 8 K [46]. Hut formation studied by high resolution low-energy electron diffraction and surface-stress-induced optical deflection approved that at deposition temperature of 500°C hut formation suddenly set in at a coverage of 3.5 ML [47]. And finally, for theoretical studies, the WL thickness and consequently the hut formation coverage are usually assumed to equal 3 ML [48]. As it is seen from the above examples, there is no unambiguous information presently about the coverage at which huts arise or, more accurately, about the thickness of the WL *M *× *N *patch on which a cluster nucleate during Ge deposition. STM studies show the WL thickness to equal 3 ML only on the average: *M *× *N *patches have slightly different thicknesses (±1 ML) around this value [18,20,32,41,49]. In this section, we determine by means of high resolution STM an accurate value of the *M *× *N *patch height at which huts nucleate at 360°C.

**Figure 3:**
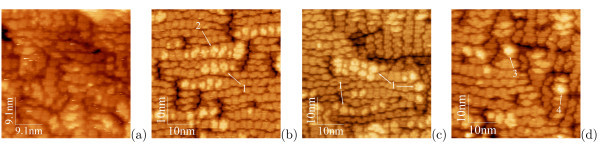
**STM images of Ge WL on Si(001) at the outset of QD array formation**: *T*_gr _= 360°C, **a ***h*_Ge _= 4.4 Å, *U*_s _= -1.86 V, *I_t _*= 100 pA, neither hut clusters nor their nuclei are observed; **b ***h*_Ge _= 5.1 Å, *U*_s _= +1.73 V, *I_t _*= 150 pA; **c ***U*_s _= +1.80 V, *I_t _*= 100 pA; **d ***U*_s _= +2.00 V, *I_t _*= 100 pA. Examples of characteristic features are numbered as follows: nuclei of pyramids (1) and wedges (2) [1 ML high over WL patchs, Figure 1] [20,32], small pyramids (3) and wedges (4) [2 ML high over WL patchs] [18,20,32,49].

Figure 3a demonstrates a typical STM micrograph of the (*M *× *N*)-patched WL (*h*_Ge _= 4.4 Å, ~3.1 ML). This image does not demonstrate any feature that might be recognized as a hut nucleus (Figure 1a-c) [32]. Such features first arise at the coverages of ~5 Å: they are clearly seen in Figure 3b-d, which demonstrate a moment when the array have just nucleated (*h*_Ge _= 5.1 Å, ~3.6 ML). However, we succeeded to find minute pyramid and wedge at this *h*_Ge _(Figure 3d)--both as small as 2 ML over the patch surface (we measure cluster heighs from patch tops)--which indicate that hut nucleation had started a little earlier.

It can be concluded from these observations that hut arrays nucleate at a coverage of ~5.1 Å (~3.6 ML) when approximately a half of patches are as thick as 4 ML. We can suppose then that huts nucleate on those patches whose thickness have reached (or even have exceeded) 4 ML.

### Morphology

#### Wetting layer reconstruction

**Figure 4:**
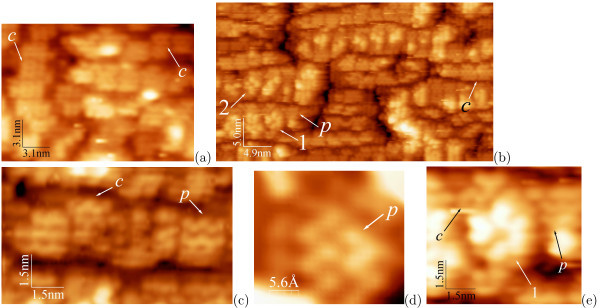
**STM images of Ge WL on Si(001)**: *T*_gr _= 360°C, the ordinary *c*(4 × 2) (*c*) and *p*(2 × 2) (*p*) reconstructions within the *M *× *N *patches are often observed simultaneously, **a ***h*_Ge _= 4.4 Å, *U*_s _= -1.86 V, *I_t _*= 100 pA, only the *c*(4 × 2) structure is resolved; **b ***h*_Ge _= 5.1 Å, *U*_s _= -3.78 V, *I_t _*= 100 pA, both *c*(4 × 2) and *p*(2 × 2) structures are revealed as well as nuclei of a pyramid (1) and a wedge (2); **c**, **d ***h*_Ge _= 6.0 Å, *U*_s _= +1.80 V, *I_t _*= 80 pA, both *c*(4 × 2) and *p*(2 × 2) reconstructions are well resolved; **e ***h*_Ge _= 5.1 Å, *U*_s _= -3.78 V, *I_t _*= 100 pA, a pyramid nucleus on the *c*(4 × 2) reconstructed patch with the adjacent *p*(2 × 2) reconstructed patch.

Evolution of WL patches during MBE is illustrated by Figure 4. In full agreement with the data of Ref. [41], both *c*(4 × 2) and *p*(2 × 2) reconstructions are observed on tops of the *M *× *N *patches in all images except for the image given in Figure 4a (*h*_Ge _= 4.4 Å) in which only the *c*(4 × 2) structure is recognized. A magnified image of the *p*(2 × 2) structure illustrating its characteristic zig-zagged shape and resolving separate upper atoms of buckled Ge dimers is given in Figure 4d.

Formation of a hut nucleus on a patch reconstructs its surface; a new formation changes the structure of the topmost layer to that specific for a particular type of nuclei, in the present case, to the structure of the pyramidal hut nucleus (Figure 4e). However, the residual *c*(4 × 2) structure still remains on the lower terrace of the patch. At the same time, the *p*(2 × 2) structure stays on the top of the adjacent patch [33].

We can conclude now that *c*(4 × 2) and *p*(2 × 2) surface structures occurring on the *M *× *N *patches should be energetically degenerate. In addition, the above observation rises a question whether there is some connection between the form of a patch top reconstruction and a species of hut that could nucleate on it or, in other words, whether the patch top reconstruction controls hut nucleation and determines its species.

#### Growth and structure of pyramids and wedges

**Figure 5:**
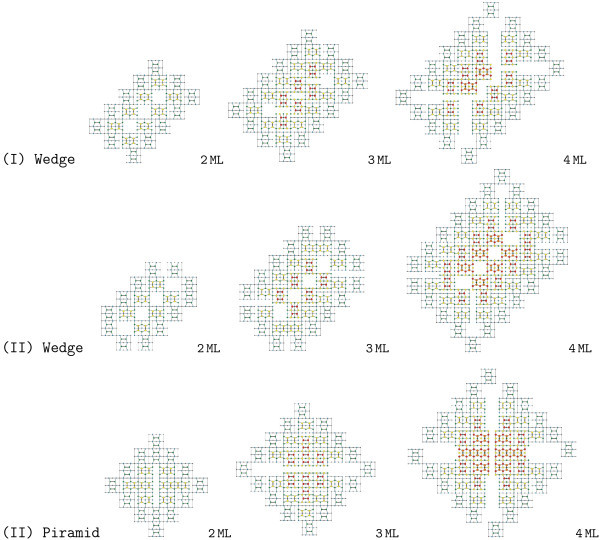
**Models of Ge hut growth**: (I) uniform addition of Ge atoms to four facets; (II) non-uniform addition of Ge atoms to facets.

Let us consider possible scenarios of hut growth after nucleation on WL. Earlier [18,20,49], we have already proposed structural models of both species of huts and briefly discussed processes and atomic models of their formation giving a few drawings with identical apexes as examples and allowing the readers to construct the missing structural schemes. However, crystallography allows one to arrive to two different solutions for wedge-like huts, and additional empirical knowledge and STM data are required to discriminate between them. Both solutions are given in Figure 5. The first scenario of growth assumes *uniform addition of Ge atoms *to all four facets of huts (follow a series number I in Figure 5). In this case, wedge-like huts have different ridge structures (the ridge width and location of atoms on it) depending on cluster height. The initial ridge structure, which form on top of 2-ML wedge reconstructing the nucleus [20,32], should then occur on the ridge every 5 ML over the nucleus, i.e., only the wedges of 2, 6, 11, etc. ML height over WL can have the same ridge structure. This contradicts our observations according to which the structure of hut apexes always remains and depends on only the hut species. Therefore, we are made to come to a different solution in which *Ge atoms are added to facets non-uniformly *(see series II for wedges and pyramids in Figure 5). In this scenario, the structures of both apexes of huts are independent of cluster heights, that agrees with experimental observations.

#### Complete cycle of Ge QD array growth at low temperatures

**Figure 6:**
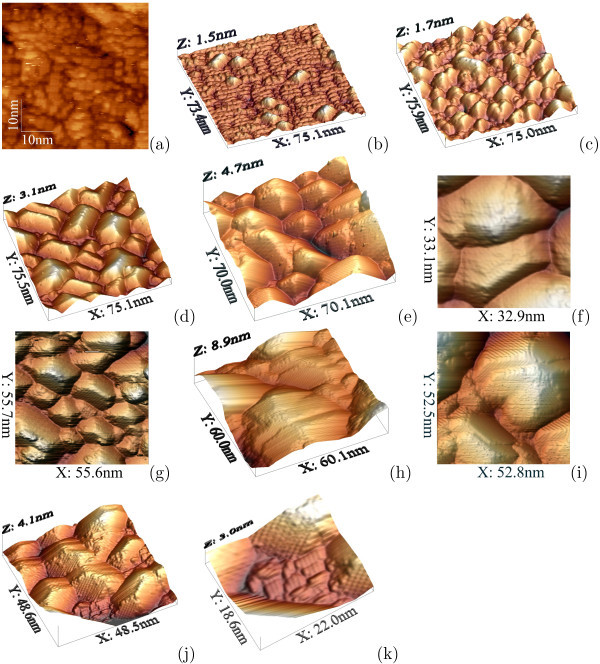
**STM images of a Ge QD dense array at different phases of its evolution from patched WL to 2D nanocrystalline layer**: *T*_gr _= 360°C, *h*_Ge _= **a **4.4 Å, before nucleation (see also Figure 3 for details of array nucleation at *h*_Ge _= 5.1 Å); **b **6 Å, growing small huts, nucleation goes on; **c **8 Å, maximum density (~6 × 10^11 ^cm^-2^); **d **10 Å, maximum uniformity, large huts start to coalesce; **e**, **f **14 Å, huts go on coalescing; **g **15 Å, 2D layer starts to form; **h**, **i**, **j**, **k **18 Å, 2D nanocrystallyne film grows, chaos of faceted hillocks and pits (**i**) is observed; however, Ge WL (*M *× *N*)-patched structure is clearly resolved on bottom of pits (**j**, **k**).

The STM images of the surfaces of the germanium layers grown at *T*_gr _= 360°C with various *h*_Ge _values are shown in Figure 6 where the evolution of the Ge layer on the Si(001) surface in the process of low-temperature MBE is seen. Hut clusters on the Ge surface have not nucleated at *h*_Ge _= 4.4 Å, and the STM image in Figure 6a exhibits only the well-known structure of the wetting layer with the *c*(4 × 2) or *p*(2 × 2) reconstruction inside *M *× *N *blocks [20,32]. The array nucleates at *h*_Ge _~ 5 Å (Figure 3) but 3D huts mainly form at higher coverages [20,33]. Hut arrays initially evolve with increasing *h*_Ge _by concurrent growth of available clusters and nucleation of new ones resulting in progressive rise of hut number density. Huts are clearly seen in Figure 6b for *h*_Ge _= 6 Å; their density and sizes increase; the number density of huts reaches maximum at *h*_Ge _= 8 Å (Figure 6c); clusters with various sizes--completely formed clusters, recently nucleated small clusters, and nuclei with a height of 1 ML over the Ge WL--are simultaneously present on the surface [20,32,33]. This array is very inhomogeneous both in the sizes of the clusters and in composition; it includes regular pyramidal and elongated wedge-shaped clusters, but wedge-shaped clusters with a large spread in the lengths dominate [18]. The array is most homogeneous at *h*_Ge _= 10 Å (Figure 6d) [50], clusters cover almost the entire surface of the wetting layer, the fraction of small clusters decreases noticeably, and large clusters begin to coalesce. At *h*_Ge _= 14 Å, most clusters coalesce near their bases (Figure 6e, f), and the free wetting layer almost disappears from the field of view of STM, but the array consists of individual clusters. At *h*_Ge _= 15 Å, the coalescence of clusters continues and a transition to the growth of a two-dimensional film of nanocrystalline germanium begins (Figure 6g). Nevertheless, the hut nucleation continues on small lawns of WL rarely preserved, surrounded by large huts, even at as high coverages as 15 Å, when virtually total coalescence of the mature huts has already happened [20]. Finally, at *h*_Ge _= 18 Å, it is seen that the array of Ge clusters disappears, and although the roughness of the surface is still pronounced, the Ge layer grows as a continuous nanocrystalline film (Figure 6h). A chaotic conglomeration of faceted hillocks and pits composes the film; steep facets appear around the pits (Figure 6i). However, Ge WL (*M *× *N*)-patched structure is clearly resolved on the bottom of pits and WL lawns (Figure 6j, k). WL appears to be a very stable formation.

The density of the wedge-shaped clusters increases when *h*_Ge _increases up to 8 Å and, then, decreases slowly, whereas the density of the pyramid-shaped clusters decreases exponentially in the process of growth of the array [18,20]. The total density of the clusters is about 3.5 × 10^11^, 5.8 × 10^11^, 5.1 × 10^11^, and 2.3 × 10^11 ^cm^-2 ^at *h*_Ge _= 6, 8, 10, and 14 Å, respectively. From the capacitance-voltage characteristics of the samples with Ge/Si(001) heterostructures, the surface densities of holes in them were earlier estimated as 3.4 × 10^11^, 7.0 × 10^11^, and 1.7 × 10^11 ^cm^-2 ^for *h*_Ge _= 6, 10, and 14 Å, respectively. These values almost coincide with the densities of the Ge clusters in arrays [51,52]. Notice also that very high terahertz conductivity was observed by Zhukova et al. [52] in the samples with *h*_Ge _= 8, 9, 10, and 14 Å, which drastically decreased for *h*_Ge _= 18 Å and was not detected at all at 6 Å and lower values of *h*_Ge_.

### CMOS compatibility

CMOS compatibility of technological processes based on Ge/Si heteroepitaxy imposes a hard constraint on conditions of all the phases of the heterostructure formation including Si wafer thermal cleaning and surface preparation to epitaxial growth. Formation of a device structure with QD arrays as a rule must be one of the latest operations of the whole device production cycle because otherwise the QD arrays would be destroyed by further high-temperature annealing. High-temperature processes during Ge/Si heterostructure formation on the late phase of chip production would, in turn, certainly wreck a circuit already formed on the crystal. Therefore, lowering of the array formation temperature down to the values of ≲450°C, as well as decreasing of the wafer annealing temperatures and times during the clean Si(001) surface preparation, is strongly required [20]. We refer to the Ge QD arrays and heterostructures based on them that satisfy this requirement as CMOS-compatible.

#### Si(001) hydrogenation as a promising way of reduction of the surface cleaning temperature

Development of a procedure of clean Si(001) surface preparation at lowered temperatures and/or by short thermal treatments is a keystone of creation of a CMOS-compatible process of nanoelectronic VLSI fabrication [18,32]. One of the ways of solving this problem is surface hydrogenation during wet chemical etching with subsequent hydrogen desorption from the surface in UHV ambient [27,53]. In this connection, an issue of surface structure after these treatments becomes a task of primary importance taking into account a possible effect of Si surface atomic-scale roughness on formation of nanostructured elements (e. g., self-assembled Ge quantum dot nucleation on wetting layer in Ge/Si(001) heterostructures [20,32,54,55]). In this section, we present data of our recent investigations conducted by means of STM and RHEED on preparation of clean Si(001) surfaces by hydrogenation and thermal desorption of hydrogen in an UHV MBE chamber after wet chemical etching by the RCA process [31].

**Figure 7:**
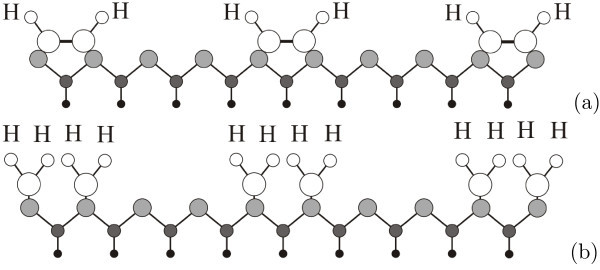
**Structure of a hydrogenated Si(001) surface**: **a **mono-hydride and **b **di-hydride.

It is known [53,56] that the temperature of surface cleaning depends on composition of etchants used for hydrogenation. Solutions based on HF, with pH varied from 2 to 7, are typically used for surface hydrogenation. A number of silicon hydrides form on the Si(001) surface by the reaction of hydrogen with Si, and the most typical ones are monohydride and dihydride (Figure 7). A fraction of dihydride on the surface grows with the increase of pH; monohydride desorbs from the surface at higher temperature [53].

**Figure 8:**
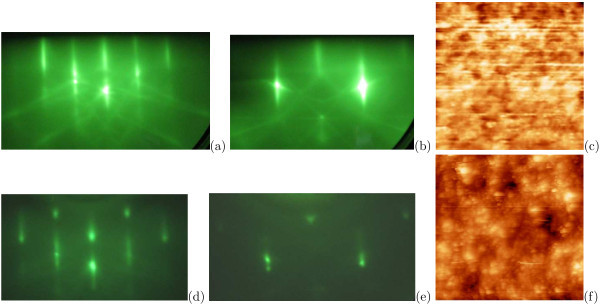
**RHEED patterns and STM images of Si:H surfaces obtained as a result of different chemical treatments**: **a**-**c **after hydrogenation in dilute HF; **d**-**f **after hydrogenation in buffered HF + NH_4_F; RHEED patterns: *E *= 10 keV, **a**, **d **[110] azimuth, **b**, **e **[010] azimuth; STM empty-state images: **c **100 × 100 nm, *U*_s _= +1.9 V, *I_t _*= 100 pA; **f **88 × 88 nm, *U*_s _= +2.0 V, *I_t _*= 100 pA.

The main results of our studies are as follows:

Explorations of *hydrogenated surfaces *[31] have evidenced that regardless of the type of solution used for surface hydrogenation, RHEED patterns correspond with unreconstructed 1 × 1 surface (Figure 8). Broad streaks with pronounced 3D-related structure form the RHEED patterns for the samples etched in HF solutions (Figure 8a, b); high intensity of the Kikuchi lines indicates that the surface is highly smooth and ordered on macroscopic scale. Visible local enhancement of signal of the RHEED patterns takes place owing to overlapping of Kikuchi lines. The shapes of the streaks corresponding to the surface well developed on the monoatomic scale (3D spots) are detected in the patterns of the samples treated in NH_4_F solutions (Figure 8d, e). According to STM data, the surface is more rough in the case of etching in the NH_4_F solutions than in the case of hydrogenation in solutions based on HF.

**Figure 9:**
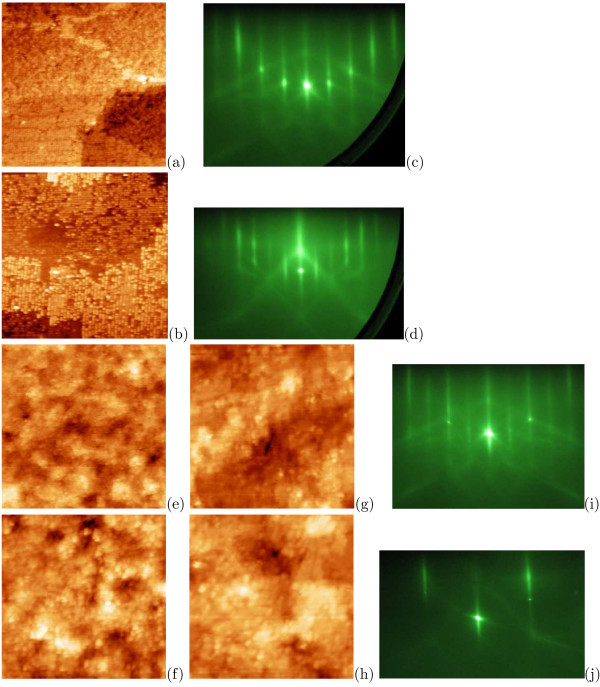
**STM images and RHEED patterns of Si:H surfaces obtained as a result of hydrogenation in dilute HF after different heat treatments**: **a**, **b**, **e**-**h **STM empty-state images; **a **650°C for 8 min, 57 × 57 nm; **b **610°C for 10 min, 41 × 41 nm; **c**, **d **corresponding RHEED patterns, *E *= 10 keV: **c **[110], **d **[010]; **e **570°C for 20 min, 101 × 101 nm; **f **550°C for 30 min, 66 × 66 nm; **g **530°C for 35 min, 41 × 41 nm; **h **500°C for 35 min, 49 × 49 nm; **i**, **j **corresponding RHEED patterns, *E *= 10 keV: **i **[110], **j **[010].

**Figure 10:**
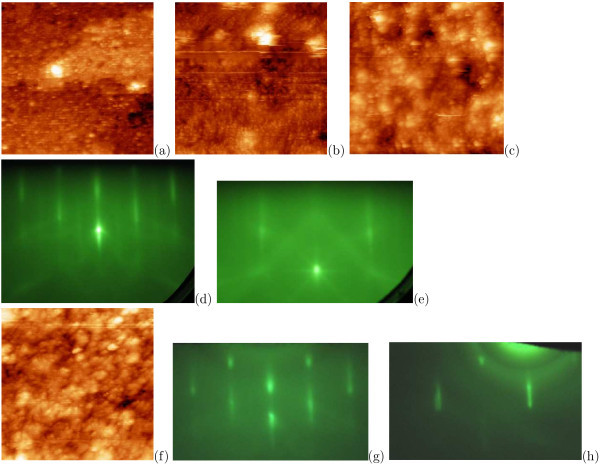
**STM images and RHEED patterns of Si:H surfaces obtained as a result of hydrogenation in NH_4_F or HF + NH_4_F solution after different heat treatments**: **a**-**c**, **f **STM empty-state images; **a **NH_4_F, 650°C for 5 min, 40 × 40 nm; **b **HF + NH_4_F, 610°C for 10 min, 56 × 56 nm; **c **NH_4_F, 610°C for 10 min, 88 × 87 nm; **d**, **e **corresponding RHEED patterns, *E *= 10 keV: **d **[110], **e **[010]; **f **NH_4_F, 550°C for 35 min, 60 × 60 nm; **g**, **h **corresponding RHEED patterns, *E *= 10 keV: **g **[110], **h **[010].

STM measurements have shown that clean Si(001) surfaces may be obtained as a result of *hydrogen thermal desorption *in the interval from 470 to 650°C [31], but their roughness depends on chemical treatment applied for hydrogenation and temperature of subsequent annealing (Figures 9, 10).

If dilute HF solutions and annealings of moderate duration at temperatures higher than 600°C are applied, smooth surfaces with monoatomic steps and wide terraces are obtained (Figure 9a-d). The *c*(4 × 4) reconstruction is observed for such samples. If the duration of annealing at the temperature higher than 600°C is increased, SiC islanding may occur on the surface. Lower temperature of annealing gives rise to formation of a rough (2 × 1)-reconstructed surface (Figure 9e-j).

Application of solutions based on NH_4_F followed by any low-temperature annealing enables obtaining of clean rough Si(001) surfaces composed by narrow and short terraces and monoatomic steps (Figure 10). The (2 × 1)-reconstructed surface forms as a result of annealing at the temperatures higher than 600°C (Figure 10d, e), 1 × 1 surface was observed after treatments at lower temperatures (Figure 10g, h). We would like to emphasize that annealing at the temperatures from 470 to 600°C results in formation of rough surfaces regardless of the applied chemical treatment (compare Figures 9e-j and 10f-h); application of solutions containing NH_4_F always results in formation of more rough surfaces in comparison with surfaces of specimens treated in dilute HF solutions (Figure 10).

Notice that the (2 × 1) RHEED patterns were observed for the hydrogenated surfaces after annealing at 800°C for 5 min and quenching [28], which were used as the reference samples with known surface structure.

It should be noted also that comparison of the above STM and RHEED data makes one infer that RHEED cannot be applied as the only method of monitoring of the surface cleaning grade and the state of dehydrogenated surfaces [31]. RHEED patterns on the hydrogenated surfaces corresponded to the 1 × 1 structure. Surfaces cleaned as a result of subsequent annealings in the temperature interval from 470 to 650°C were (1 × 1) or either (2 × 1) or *c*(4 × 4)-reconstructed. Hence, in some cases, a type of the RHEED pattern did not change after thermal desorption of hydrogen; however, forms of the patterns, which corresponded to the 1 × 1 structure, were different before and after annealings.

As of now, we suppose that clean Si(001) surfaces applicable for MBE formation of Ge/Si(001) heterostructures can be obtained at low temperatures (as low as 470°C). However, it is not excluded that further lowering of temperature of the clean Si(001) surface preparation is possible, perhaps down to the temperatures as low as 400°C [53]. In the latter case, Si weak flux and formation of a buffer layer may be useful to prepare a good enough Si(001) surface before Ge deposition.

**Figure 11:**
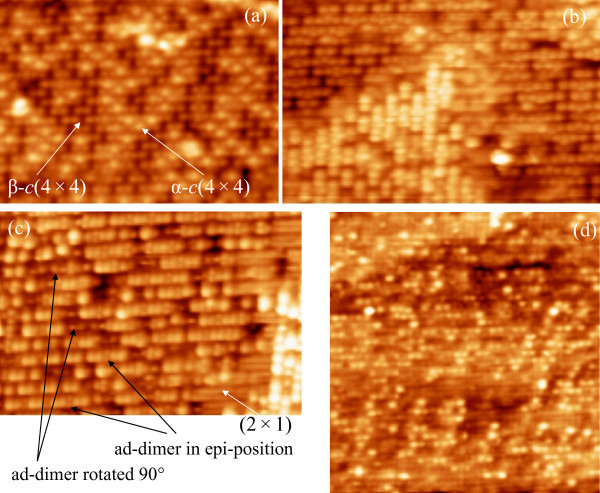
**STM images of the Si(001)-*c*(4 × 4) surface**: **a **empty states, 19 × 14 nm, *U*_s _= +2.5 V, *I_t _*= 120 pA; **b **empty states, 19 × 12 nm, *U*_s _= +2.0 V, *I_t _*= 120 pA; **c **filled states, 22 × 15 nm, *U*_s _= -3.9 V, *I_t _*= 150 pA; **d **filled states, 19 × 20 nm, *U*_s _= -3.9 V, *I_t _*= 150 pA. As it is clearly observed in **a**, the structure is composed by a mixture of the *α*-c(4 × 4) and *β*-c(4 × 4) modifications; it is seen in **c **that the *c*(4 × 4) and (2 × 1) reconstructions coexist on the surface; location of dimers forming the *c*(4 × 4) structure with respect to the dimers of the (2 × 1) structure is also seen; ad-dimers in both epitaxial and non-epitaxial orientations are seen in **c**. The *β*-*c*(4 × 4) modification prevails in **d **which is only partially occupied by *c*(4 × 4).

Concluding this section, let us briefly consider the morphological peculiarities of the *c*(4 × 4)-reconstructed surface shown in Figure 9a, b. Figure 11 presents magnified STM images of this surface. A structure observed in the images represents a mixture of *α*-*c*(4 × 4) and *β*-*c*(4 × 4) modifications [57,58]; (2 × 1) and *c*(4 × 4)-reconstructed domains coexist on the surface (Figure 11c); location of dimers forming the *c*(4 × 4) structure with respect to the dimers of the (2 × 1) structure is also seen; ad-dimers in both epitaxial and non-epitaxial orientations are seen in Figure 11c. The *β*-*c*(4 × 4) modification prevails on the surface shown in Figure 11d, which is only partially occupied by *c*(4 × 4). It is seen that the presented data are in a good agreement with the model of the *c*(4 × 4) structure proposed by Uhrberg et al. [57,58].

## Conclusions

### Is 600°C a fundamental value of temperature for Si and Ge (001) surfaces?

Concluding the article, we would like to draw the reader's attention to the fact that many of the processes described above or in the cited articles have some critical temperature close to 600°C. Thus, the phase transition between 2 × 1 and *c*(8 × 8) reconstructions occurs around this temperature [19,28]. Exploration of dehydrogenation of the Si:H samples shows that clean surfaces obtained by annealing at the temperatures *>*600°C are formed by wide terraces with monoatomic steps; the *c*(4 × 4) reconstruction appears at these temperatures [57]. Annealing at the temperatures *<*600°C results in formation of rough surfaces composed by narrow and short steps. Ge QD arrays deposited by MBE at the temperatures ≲ and ≳600°C also strongly differ in both cluster compositon and nucleation. Bimodal hut arrays form at low temperatures, whereas arrays grown at high temperatures are composed by pyramids and domes. The low-temperature clusters nucleate by formation of strictly determined 2D structures composed by dimer pairs and longer chains [20,32]. There are two alternative scenarios of cluster formation at high temperatures: (i) similarly to the low-temperature nucleation of pyramids and (ii) by {105}-faceting of the Ge shapeless heaps. An assumption arises from these examples that the critical temperatures do not coincide accidently, but some changes happen in the processes of migration of Si and Ge adatoms over the (001) surface around 600°C.

### Summary

In summary, issues of morphology, nucleation, and growth of Ge cluster arrays deposited by ultrahigh vacuum molecular beam epitaxy on the Si(001) surface are considered in the article. Difference in nucleation of quantum dots during Ge deposition at low (≲600°C) and high (≳600°C) temperatures is studied by high resolution scanning tunneling microscopy. The atomic models of growth of both species of Ge huts--pyramids and wedges--are proposed. The growth cycle of Ge QD arrays at low temperatures is explored. A problem of lowering of the array formation temperature is discussed with the focus on CMOS compatibility of the entire process; a special attention is paid upon approaches to reduction of treatment temperature during the Si(001) surface pre-growth cleaning, which is at once a key and the highest-temperature phase of the Ge/Si(001) quantum dot dense array formation process. The temperature of the Si clean surface preparation, the final high-temperature step of which is, as a rule, carried out directly in the MBE chamber just before the structure deposition, determines the compatibility of formation process of Ge-QD-array based devices with the CMOS manufacturing cycle. Silicon surface hydrogenation at the final stage of its wet chemical etching during the preliminary cleaning is proposed as a possible way of efficient reduction of the Si wafer pre-growth annealing temperature.

## Abbreviations

AES: Auger electron spectroscopy; CMOS: complementary metal-oxide semiconductor; CZ: Czochralski or grown by the Czochralski method; MBE: molecular beam epitaxy; ML: monolayer; PD: pairs of dimers; QD: quantum dot; RCA: Radio Corporation of America; RHEED: reflected high-energy electron diffraction; RS: rebonded step; SIMS: secondary ion mass spectroscopy; STM: scanning tunneling microscope; XPS: X-ray photoelectron spectroscopy; WL: wetting layer; UHV: ultra-high vacuum.

## Competing interests

The authors declare that they have no competing interests.

## Authors' contributions

VY conceived of the study and designed it, processed images and performed data analysis, and took part in discussions and interpretation of the results; he also supervised and coordinated the research projects. LA participated in the design of the study, carried out the experiments, performed data analysis, and took part in discussions and interpretation of the results; she proposed the structural models; she also supervised the research project (∏2367) and led the development of surface cleaning processes.

## Endnotes

^a^The ideas underwritten in this paragraph have already been proposed by us [59] and A. V. Dvurechenskii.

^b^In addition, an analytical chamber equipped with secondary ion mass spectroscopy (SIMS), Auger electron spectroscopy (AES), and X-ray photoelectron spectroscopy (XPS) is also available in the instrument. A Knudsen effusion cells for layer doping by boron during deposition is installed in the MBE chamber, but it was not used in the described experiments.

^c^We express our acknowledgment to the anonymous colleague who proposed this explanation.
